# Evaluation of heroin-assisted treatment in Norway: protocol for a mixed methods study

**DOI:** 10.1186/s12913-024-10767-w

**Published:** 2024-03-29

**Authors:** Lars Henrik Myklebust, Desiree Eide, Espen A. Arnevik, Omid Dadras, Silvana De Pirro, Rune Ellefsen, Lars T. Fadnes, Morten Hesse, Timo L. Kvamme, Francesca Melis, Ann Oldervoll, Birgitte Thylstrup, Linda E.C. Wusthoff, Thomas Clausen

**Affiliations:** 1 Norwegian Centre for Addiction Research, Faculty of Medicine, University of Oslo, Kirkevegen 166, Building 45, NO-0407 Oslo, Norway; 2 Bergen Addiction Research Group, Department of Addiction Medicine, Haukeland University Hospital, P.b 1400, NO-5021 Bergen, Norway; 3 Department of Global Public Health and Primary Care, University of Bergen, P.b 7804, NO-5020 Bergen, Norway; 4 Section for Clinical Addiction Research, Oslo University Hospital, P.b 4950 Nydalen, NO-0424 Oslo, Norway; 5 Centre for Alcohol and Drug Research, Aarhus University, Bartholins Allé 10, DK-8000 Aarhus, Denmark; 6 Department of Physiology and Pharmacology “V. Erspamer,” La Sapienza, University of Rome, P. Ie Aldo Moro 5, 00185 Rome, Italy

**Keywords:** Heroin assisted treatment, Opioid maintenance treatment, Protocol, Health services, Mixed methods, Longitudinal

## Abstract

**Background:**

Opioid agonist treatment (OAT) for patients with opioid use disorder (OUD) has a convincing evidence base, although variable retention rates suggest that it may not be beneficial for all. One of the options to include more patients is the introduction of heroin-assisted treatment (HAT), which involves the prescribing of pharmaceutical heroin in a clinical supervised setting. Clinical trials suggest that HAT positively affects illicit drug use, criminal behavior, quality of life, and health. The results are less clear for longer-term outcomes such as mortality, level of function and social integration. This protocol describes a longitudinal evaluation of the introduction of HAT into the OAT services in Norway over a 5-year period. The main aim of the project is to study the individual, organizational and societal effects of implementing HAT in the specialized healthcare services for OUD.

**Methods:**

The project adopts a multidisciplinary approach, where the primary cohort for analysis will consist of approximately 250 patients in Norway, observed during the period of 2022–2026. Cohorts for comparative analysis will include all HAT-patients in Denmark from 2010 to 2022 (*N* = 500) and all Norwegian patients in conventional OAT (*N* = 8300). Data comes from individual in-depth and semi-structured interviews, self-report questionnaires, clinical records, and national registries, collected at several time points throughout patients’ courses of treatment. Qualitative analyses will use a flexible inductive thematic approach. Quantitative analyses will employ a wide array of methods including bi-variate parametric and non-parametric tests, and various forms of multivariate modeling.

**Discussion:**

The project’s primary strength lies in its comprehensive and longitudinal approach. It has the potential to reveal new insights on whether pharmaceutical heroin should be an integral part of integrated conventional OAT services to individually tailor treatments for patients with OUD. This could affect considerations about drug treatment even beyond HAT-specific topics, where an expanded understanding of why some do not succeed with conventional OAT will strengthen the knowledge base for drug treatment in general. Results will be disseminated to the scientific community, clinicians, and policy makers.

**Trial registration:**

The study was approved by the Norwegian Regional Committee for Medical and Health Research Ethics (REK), ref.nr.:195733.

## Background

Opioid use disorder (OUD) is a major global health concern with an estimated caseload of 31.5 million in 2022 [1]. It is frequently related to infectious diseases from injection-based drug use, psychiatric disorders, deterioration of social relations, reduced workforce participation, and a tenfold increase in crude all-cause rate of mortality [2]. The treatment and care for patients with OUD has gradually developed from an initial emphasis on abstinence and withdrawal management, to regular prescriptions of opioid agonists for maintenance treatment (OAT) [3].

Half a century after the first initiatives of prescribing methadone for OUD in a regular manner [4, 5] OAT now has a strong evidence-base [6]. Overall, it contributes to a substantial reduction in mortality, general health benefits, and reduced use of illicit drugs and criminal activity [6–9]. Still, not all individuals find conventional OAT sufficiently attractive over time, and cycles of dropout and re-entering are ongoing challenges in these programs [10–12]. A variable retention rate of 20–84% has been observed [13]. Among the efforts to improve the inclusion of patients in OAT is the introduction of more diverse medication options, such as rapid-onset, short-acting injectable pharmaceutical opioids such as heroin [14].

The use of medical grade heroin (diacetylmorphine) in treating OUD has been applied in England since the 1920s, originally as hand-out prescriptions to take home [15, 16]. Initiatives to incorporate it into more regular OAT started in Switzerland in 1994, with promising results [17, 18]. Now, three decades later and after clinical trials from several European countries and Canada, the body of research suggests that heroin-assisted treatment (HAT) is beneficial for a sub-selection of patients in regard to health outcomes and reductions in use of illicit drugs and criminal behavior [19–21]. The results are less clear for longer-term outcomes such as mortality [6, 19].

Still, HAT remains politically controversial [22], and reduced illicit heroin use and criminal behavior may not be compelling arguments for its efficacy. Rather, as for any other medical treatment its impact may better be assessed by patients’ improvement in quality of life, everyday level of function, and mortality [23].

Although newer studies suggests that take home doses are a feasible and safe alternative for patients deemed suitable [24, 25], medical heroin is typically administered under rigorous and comprehensive medical supervision due to the risk of serious adverse events and diversion [26]. Studies on cost effectiveness suggest both excessive expenses and inconclusive results when compared with methadone treatment, which are possibly dependent on methodological issues and poor consideration of the mechanisms involved [20, 27, 28].

Additionally, most of the research on the effectiveness of HAT originates from randomized clinical trials which may have limitations concerning the understanding of long-term outcomes and the mechanisms behind [23]. Thus, the main contribution of HAT may lie in the engagement of a high-risk population in utilization of health- and social services over time, like the more conventional options of OAT [23, 29]. A more comprehensive view of outcomes beyond the mere quantity and frequency of drug use and criminal behaviour can provide crucial information about the mechanisms responsible for treatment effectiveness, and its possible impact on other clinically and socially relevant parameters [30].

The current Norwegian HAT study is presented in this context. The study is part of a clinical project by the Norwegian Directorate of Health, with the aim to evaluate the implementation of HAT into the national OAT services. It is based on a model from Denmark where the use of medical heroin was introduced in 2010, following the British “RIOTT” line of test trials from 2005 [31]. Denmark currently has five clinics as permanent parts of the national healthcare system, although a limited amount of research has been published from this model [32].

### The Norwegian HAT-project

OAT programs based on prescription of methadone and buprenorphine has in various forms been integrated into the Norwegian health and social services-system since 1997 [33]. In the spring of 2020, the Norwegian Directorate of Health introduced a time-limited, clinically based project on the use of pharmaceutical heroin in the specialist healthcare services. Based on a day-center model, treatment is offered at two designated clinics in the largest Norwegian cities of Oslo and Bergen. The clinics consist of injection sites and medical personnel for the administration of pharmaceutical heroin twice a day, in combination with a take-home oral overnight dose of slow-releasing opioid-agonist such as methadone or morphine. Take-home doses of heroin are not granted, and patients must attend daily all year around. Psychosocial services and support are also offered [34]. Patients are referred from other services of substance use disorder treatment, specialist healthcare services or general practitioners. Criteria for admission are ongoing OUD with at least one former attempt of conventional OAT, being over 18 years of age and with general competency of consent. Exclusion criteria are severe mental disorders with reduced competency of consent, pregnancy, or repeated violent behavior.

The Norwegian Centre for Addiction Research (SERAF) at the University of Oslo was granted the research-based evaluation of the HAT project in 2021. The study will be conducted together with Section for Clinical Addiction Research (RusForsk) at Oslo University Hospital, Bergen Addiction Research Group (BAR) at Haukeland University Hospital in Bergen, Centre for Alcohol and Drug Research (CRF) at Aarhus University in Denmark, and the Norwegian user organization proLARNett.

### Study aims

The primary aim of the research project is to examine the effects from implementing HAT in Norway for individual patients and for the health services organization. A secondary aim is to compare these findings with the Danish HAT program.

Based on the Norwegian Directorate of Health’s specifications in the project proposal, the study will cover the following thematic areas:

1. Explore the attitudes, experiences and challenges of HAT as perceived by patients, their relatives, and clinical staff.
2. Describe changes in mental and physical health among patients receiving HAT, and in what way it is associated with outcomes such as quality of life, utilization of health- and social services, social reintegration, criminal behavior and use of illicit drugs.
3. Report any serious adverse events and incidents at treatment initiation, during treatment, and after discharge from HAT.
4. Perform an economic evaluation of the program with associated clinical benefits and societal costs.
5. Evaluate the organizational processes involved in the implementation of HAT in Norwegian specialist healthcare services, and the eventual impact from HAT on OUD patients’ utilization of conventional OAT.
6. Additional research relevant to HAT that is not explicitly outlined in the proposal (may require additional approvals from the Norwegian Regional Committee for Medical and Health Research Ethics.)

The themes were operationalized into six work packages, with corresponding research questions and data sources (shown in Table 1).

**Table 1:** Overview of work packages

	Work package	Data source	Research questions
1	Attitudes and experiences of HAT	In-depth and semi-structured interviews with patients, family members and staff	What are the patients’ and staff’s experiences and views of HAT? What may be the effective and ineffective elements of HAT? What are the patients’ uses of other health and social services when in HAT? What are the patients’ preferences and views of medications in HAT?
2	Health, social, and treatment outcomes	Questionnaires Clinical records Registries	Does HAT affect patients’ physical and mental health over time? Does HAT affect use of health and social services and quality of life? Does HAT affect the use of health and social services? Does HAT affect criminal activities? Does HAT affect illicit drug use?
3	Serious adverse events	Clinical records	Does HAT affect frequencies of serious adverse events and overdoses among patients? How do these events differ from those among patients in standard OAT? What are the clinical circumstances and outcomes for serious adverse events that occur in the HAT clinic?
4	Cost-benefit evaluation	Clinical records Registries Key-account figures	What is the cost-effectiveness of HAT? Does HAT affect societal costs related to social benefits and crime? What is the cost of implementing HAT nationally in Norway?
5	Process evaluation	Interviews and questionnaires with patients, staff, and administrators	Are there critical organizational and structural elements of HAT? What are the barriers and success factors for implementing HAT in Norway? Is there a dynamic relationship in the flow of patients between HAT and standard OAT? Which patients are reached by HAT? How can HAT be implemented in the national health services?
6	Others	Additional data specified in further protocols	What are the pharmacological and subjective effects of heroin on patients, compared with other OAT medications?

## Methods and design

The project is a multi-dimensional study, involving an array of methodological approaches and data sources. The main part is a prospective cohort study of all Norwegian HAT patients, compared with the cohorts of all Danish HAT patients and Norwegian patients in conventional OAT.

### Study populations and size

The primary target group is all patients enrolled in the two HAT clinics in Oslo and Bergen during the period 2022–2026, with an expected total sample size of *N* = 250. Based on earlier findings, the ratio of men to women is expected to be 4:1, with an age distribution of 27–60, presenting multiple substance use disorders. As the study is based on the total clinical population, representation will be determined by its demographics, with no exclusion of genders or ethnic minorities. The patients who have applied to but have not been accepted for HAT will be used for comparison, with an expected sample size of 100.

Comparative data from the Danish cohort will be drawn from the comprehensive dataset at Aarhus University from 2010 and onwards, with a sample size of approximately 500 [35]. Likewise, the comprehensive dataset at SERAF on the cohort of Norwegian patients in conventional OAT from 2003 has an approximate sample size of 8300.

### Data sources

Data on the primary cohort of Norwegian HAT patients will be based on a prospective collection of both qualitative and quantitative variables from treatment inclusion and throughout the project period. For the cohorts of Danish patients, of Norwegian patients that have been referred to but not granted HAT, and of Norwegian patients in conventional OAT, data are mainly based on national registries.

### In-depth and semi-structured interviews and observation

The qualitative part of the project includes individual in-depth and semi-structured interviews with patients and relatives on their views and experiences with HAT, and focus group interviews with staff concerning implementation, clinical and legal aspects of the project. Semi-structured interview protocols have been developed by the project group and user representatives. Interviews will include 25–35 patients and 10–20 family members, conducted by a team of researchers and user representatives at 1, 6, 18 months after patients enter treatment, and with relatives after 4 and 12 months. Focus group interviews with staff will be conducted at 3, 9 and 18 months. Further, the clinic managers are being interviewed at several timepoints from the planning of the clinics and throughout the duration of the project.

For insights into clinic aspects not identified through interviews, researchers will conduct participant observation in the clinics over several periods of 1–2 weeks throughout the study.

### Questionnaires

The quantitative part of the project will use similar questionnaires to preceding projects involving patients in conventional OAT. These will evaluate changes in physical and mental health, personal economy, utilization of social services, criminal behavior and illegal drug use by repeated measures administered at inclusion, by 3, 6 and 12 months of treatment, and thereafter yearly (24, 36 and 48 months). Staff are asked to complete a separate questionnaire if a patient leaves treatment.

### Clinical records

Information will also be obtained from the individual patient’s routine clinical records on variables such as main vital signs, nutritional status, cognitive function and mental health, medication, and comorbidities, as well as more HAT-specific variables such as adverse events, dosage, and administration routes of the pharmaceutical heroin.

### Central register databases

Nordic national registers are an important and useful source for epidemiological and healthcare services research, including the study of substance use disorders [36–43]. The project will utilize databases from national registries in both Norway and Denmark to describe the cohorts and to monitor the changes and outcomes in a wider context. Currently, one study has explored the use of the Short Form (SF-36) Health Survey in patients enrolled in the Danish HAT database, finding support for the structural and external validity for its use in HAT [44].

Table 2 gives an overview of the relevant Norwegian and Danish register databases along with their relevant variables.

**Table 2:** Central register databases, Norway and Denmark

	Norway	Denmark
Treatment and health services	**National quality register for the treatment of harmful substance uses or addiction (KVARUS)** Register on specialist drug treatment-services (TSB), containing information about patient’s health and life situation, clinical variables, patient satisfaction and other outcomes after treatment.	**Registry of Drug Users receiving Treatment (SIB)** Register of patients referred to inpatient and outpatient drug-treatment services. Containing clinical variables, type of medication, admission and discharge dates, demographic information.
		**The National Register on Treatment with Heroin and Methadone (IHM)** Case registry of patients receiving treatment with injectable heroin or methadone. Includes variables on the courses (dates of admission, injection/tablet modality), as well as self-reported information about substance use, risk behavior, physical and mental illnesses, social burden, and crime.
		**Danish Registration and Information System (DanRIS)** Register from 2000 to 2010 and includes data on treatment episodes in residential rehabilitation.
Health status and health-care utilization	**Norwegian Patient Register (NPR)** National register on all patients in specialist psychiatric and somatic health care. Contains variables on diagnoses (ICD-10 codes), and modes time of treatment, and demographic information.	**Danish National Patient Registry (LPR)** National register of patients in somatic and psychiatric hospital care. Contains variables on diagnoses (ICD-10 codes), and modes time of treatment, and demographic information.
	**Norwegian Prescription Database (NorPed)** National prescriptions database through pharmacies for all Norwegian patients in both primary and specialist care.	**Danish National Prescription Register (DNPR)** National prescription database for all danish patients through both pharmacies and health institutions such as hospitals and drug treatment centers.
	**Norwegian Register on Traumas and Injuries (NTR)** Register with information on date and cause of physical trauma and accidents, death, and basic demographic variables.	**Health Insurance Statistics** Register on the consumption of health care services within the primary public health care sector.
	**The Database on Control and Payment of Public Health Reimbursements (KUHR)** Register containing information on public health reimbursements in primary and specialist health care services, profession of health personnel, type of treatment, dates and locations, and diagnosis.	
Mortality and causes of death	**The Norwegian Cause of Death Registry (DÅR)** The official cause of death statistics for Norway, including ICD-10-diagnosis, time, and place, causes and certificates of death.	**Cause of Death Registry (DAR)** The official cause of death statistics for Denmark, including ICD-10-diagnosis, time, and place, causes and certificates of death
Demographic and socioeconomic variables	**National Population Register (Norwegian Tax Administration)** Detailed information on all persons currently or have been residents in Norway. The register contains a wide range of socio-demographic variables, such as gender, date of birth, country of origin, current address, family relationships and civil status.	**Population Register (BEF)** Detailed information on all persons currently or has been residents in Denmark. The register contains a wide range of socio-demographic variables, such as gender, date of birth, country of origin, current address, family relationships and civil status.
	**Statistics Norway (SSB)** National register of all citizen’s education, employment status, income, and social security benefits (e.g., disability pension, welfare support).	**Statistics Denmark (DST)** National register of all citizen’s education, employment status, income, and social security benefits (e.g., disability pension, welfare support).
Criminal activity	**Central Criminal Registry of Norway (STRASAK)** Information about reported criminal offenses, their date and type as well as records of convictions and imprisonments.	**Danish crime registers (KRSI and KRAF)** Information about reported criminal offenses, their date and type as well as records of convictions and imprisonments.

### Additional studies

Currently, the only planned sub-study is on the pharmacokinetics of heroin and its metabolites, and its subjective effects on patients. Despite its widespread use, the pharmacology of heroin remains poorly understood [45–47]. A subsample of patients will therefore be invited to participate in this observational study with post-administration blood samples collected at different time points, with analysis of the concentration of heroin and its metabolites together with scales of subjective experience. The study has been granted separate approvals from Norwegian Regional Committee for Medical and Health Research Ethics.

### Analysis strategy

Exploration and analysis of data will be both by qualitative and quantitative strategies, for individual patients and at the organizational level.

#### Qualitative

Treatment satisfaction of patients is particularly significant to the project and is often dependent on the context of factors such as staff, management, and clinical environment [48, 49]. Qualitative analyses are widely considered valuable for description of phenomena and hypothesis generation, taking into consideration the natural context in which people and organizations function [50]. Transcribed interviews will be coded following the principles of a flexible inductive thematic analysis and multidimensional approach [51].

#### Quantitative

Given the large amount and comprehensive nature of the data, variables of interest will vary in levels of measurement and distribution, so parametric and non-parametric tests will be used accordingly.

Presentation of cohorts will include descriptive statistics by basic parameters such as mean or medians, standard deviations and ratios, and bivariate analyses by ANOVA and Chi-Square tests. Various advanced methods such as survival analysis and logistic and linear regression modeling will be applied based on the type and distribution of dependent variables and co-variates. To avoid ecological fallacy and nested dimensions, multi-level methods will be applied for analyses of patients in relation to services’ organization [52]. Given the longitudinal design and to address the repeated measurements and correlated data, linear mixed models (LMM) (random intercepts or random slope models) will be used for person-specific effects, and marginal models like Generalized Estimating Equations (GEE) for population effects.

A theoretical sample size for statistical power will not be calculated because the study is based on the total clinical population available. For analyses of discrete and possible repeated events such as the number of criminal acts or medical prescriptions, statistical power will most likely be sufficient even with a restricted number of individuals. For analyses where the proportion of patients to number of variables may imply low statistical power, stratification of the study-population and restrictions to the number of covariates in the multivariate models will be applied.

### Economic evaluations

Health economics and methods of cost-effectiveness analysis can guide decision makers, but at the same time they intrinsically rely on sets of politically and administratively determined rules and contexts [53]. In general, the cost-effectiveness of a treatment is intended to reflect the difference between the recourse’s opportunity costs (medical heroin) and those of the foregone or conventional alternative, to capture a broader set of values beyond the scope of mere financial costs [54].

Initially, for operating costs a three-step, top-down methodology used and refined by a former healthcare services project will be applied, where total costs are distributed on service units and units of treatment for individual patients [55].

For cost-effectiveness analyses of healthcare interventions, outcome is often measured in quality-adjusted life years (QALYs) for individual patients, in number of accidents or fatal incidents, or as societal costs associated with patients’ level of functioning and societal (criminal) behavior [56]. This will readily apply to the project and is in line with the national Norwegian recommendations for evaluation of new health interventions [57, 58]. The relationships between HAT and various forms of criminal behavior (both property crime and illegal drug offences), labor market attachment, income and drug expenditures are also unclear and possible subjects for investigation during the project [20].

The data for all analyses will come from key account figures and relevant variables already obtained in the project.

## Discussion

The main strength of the study comes from its clinical and longitudinal approaches. The use of patient-interviews combined with clinical records, self-report data and register-based information will enhance the analyses and may uncover important associations between the individual patients, treatment, and the organizational level of healthcare services. The results are therefore expected to address aspects of HAT that may contribute to the development of clinical services and individually tailored treatments for OUD.

Study limitations are mainly related to the designs’ limitation for isolation of the effects from HAT on the outcome variables. Although valuable associations often have been suggested by longitudinal ecological studies, this limited possibility of unbiased causal inference remains a major weakness of both epidemiological and cohort designs [59]. Consequently, analyses will be cautiously interpreted within the context of previous findings, as well as patient and staff experiences. The triangulation of different types of data sources and cohorts, with the use of multivariate analysis and modeling might nevertheless provide more nuanced insights than currently exist.

Also, socially desirable bias concerning self-report questionnaires may be inherent in all self-reported outcomes [60]. This will apply to the study, as patients in the Norwegian cohort are possibly aware that the prospects of HAT may depend on the results from the study.

The sample of patients in the main cohort might also not be representative of individuals with OUD who do not seek the HAT option for reasons related to the study outcomes, such as social deprivation and isolation, behavioral misconduct, and incarceration [61, 62]. Comparison with patients not granted access to the HAT-treatment may partly address this, although not to a full extent.

Lastly, the results will emerge in the context of a Nordic cultural and political system with healthcare reimbursements, insurance models and legal aspects that may limit their generalizability to other countries and societies. Given a cautious interpretation, the project may nonetheless be considered relevant to populations where OAT is used, and a wide range of medications are potentially provided.

Results from this project have the potential to identify new insights of value to patients, healthcare personnel, service administrators and policy makers as to whether an option for pharmaceutical heroin could be implemented as a conventional part of OAT services. We believe that the results will suggest future themes for research within the field of HAT with a potential for individually tailored treatment and care for individuals with OUD. This could affect considerations about drug treatment even beyond HAT-specific topics, where an expanded understanding of why some patients do not succeed with conventional OAT or specific OAT medications will strengthen the knowledge base for drug treatment in general.

## Data Availability

Data sharing is not applicable to this article as no datasets are currently completed or analyzed. The data that support the eventual findings of this study are available from both national registries, individual health journals and the project-specific database, but restrictions apply to the availability which are under license for the current study. Data may be available from the authors upon reasonable request and dependent on permissions from the Norwegian Regional Committee for Medical and Health Research Ethics. All information on subjects will be stored in the University of Oslo's secure services for sensitive data (TSD). Files for analysis will not contain directly identifying information of patients. Data will be stored in a non-identifiable way for 15 years after the end of the project.

## Abbreviations

OAT: Opioid agonist treatment
OUD: Opioid use disorder
HAT: Heroin assisted treatment
GEE: Generalized Estimating Equations
LMM: Linear mixed models
QALYs: Quality-Adjusted Life Years
REK: Norwegian Regional Committee for Medical and Health Research Ethics
SERAF: Norwegian Centre for Addiction Research, Oslo
RusForsk: Section for Clinical Addiction Research, Oslo
BAR: Bergen Addiction Research Group, Bergen
CRF: Centre for Alcohol and Drug Research, Aarhus
proLARnett: Norwegian User-union
